# Reviewed article: Souza MLP. Hospital mortality among psychiatric inpatients: a cross-sectional study, Brazil, 2023. Epidemiol Serv Saude. 2026:35;e20250668

**DOI:** 10.1590/S2237-96222026v35e20250668.a

**Published:** 2026-02-09

**Authors:** Cintia de Azevedo-Marques Périco

**Affiliations:** 1 Centro Multidisciplinar de Estudos Clínicos, São Bernardo do Campo, SP, Brazil Centro Multidisciplinar de Estudos Clínicos São Bernardo do Campo SP Brazil

## Peer review A

## Questionnaire

Does the manuscript contain new and significant information to justify publication? Yes

Does the Abstract (Summary) clearly and accurately describe the content of the article? Yes

Is the problem significant and concisely stated? Yes

Are the methods described comprehensively? Yes

Are the interpretations and conclusions justified by the results? Yes

Is adequate reference made to other work in the field? Yes

## Manuscript Structure

Length of article is: Adequate

Number of tables is: Adequate

Number of figures is: Adequate

Please state any conflict(s) of interest that you have in relation to the review of this paper. None

Interest: Excellent

Quality: Good

Originality: Excellent

Overall: Excellent

Recommendation: Minor Revision

## Comments

Reviewer Comments - Suggestions for Revision

The manuscript addresses a highly relevant topic in Brazilian public health, with significant potential to contribute to the ongoing debate regarding the revision of the National Mental Health Policy. The analysis of in-hospital mortality among individuals with mental disorders-particularly with stratifications by race/ethnicity and hospital type-is both timely and pertinent.

However, a few points should be addressed to improve the manuscript:

1. Association between higher mortality and white patients:

Although the finding is statistically robust, the discussion lacks depth and substantiation. The proposed explanation-that particularly vulnerable non-white individuals may face such significant barriers to accessing hospital services that they are underrepresented in the dataset-is plausible, but seems to contradict the finding that most hospitalizations occurred among non-white individuals. This contradiction should be further explored and clarified. A more nuanced and critically grounded interpretation is recommended, explicitly acknowledging data limitations and considering alternative hypotheses.

2. Racial disparities and structural racism:

The discussion presents an important perspective on structural racism and its impact on healthcare access and outcomes in mental health. However, it is recommended that the argument be more clearly structured, distinguishing between empirical findings, plausible hypotheses, and remaining knowledge gaps. This will allow for a more balanced and scientifically grounded discussion.

3. Higher mortality in general hospitals compared to psychiatric hospitals:

The finding of higher mortality in general hospitals is unexpected, considering the well-documented precarious conditions in Brazilian psychiatric institutions. While the explanation offered is reasonable-pointing to the burden of comorbidities and lack of integration between clinical and psychiatric care-it could be strengthened by incorporating additional care-related factors, as briefly mentioned by the authors themselves. For example, the stigmatization of psychiatric patients in general hospital settings, the shortage of psychiatrists in these institutions, and the lack of training among multidisciplinary teams could be discussed. Including these aspects, supported by relevant literature or health system data, would reinforce the argument regarding systemic gaps in integrated care.

Final remarks:

This study has scientific merit and public health relevance in the field of mental health, especially in contexts involving the formulation and reassessment of public health policies. The comments above aim to enhance the coherence and interpretive strength of the manuscript without compromising the validity of its findings.

I remain available for any further clarification or contribution.

